# CLASH: Complementary Linkage with Anchoring and Scoring for Heterogeneous biomolecular and clinical data

**DOI:** 10.1186/s12911-016-0315-2

**Published:** 2016-07-25

**Authors:** Yonghyun Nam, Myungjun Kim, Kyungwon Lee, Hyunjung Shin

**Affiliations:** 1 Department of Industrial Engineering, Ajou University, Wonchun-dong, Yeongtong-gu, Suwon 443-749 South Korea; 2 Department of Digital Media, Ajou University, Wonchun-dong, Yeongtong-gu, 443-749 Suwon South Korea

## Abstract

**Background:**

The study on disease-disease association has been increasingly viewed and analyzed as a network, in which the connections between diseases are configured using the source information on interactome maps of biomolecules such as genes, proteins, metabolites, etc. Although abundance in source information leads to tighter connections between diseases in the network, for a certain group of diseases, such as metabolic diseases, the connections do not occur much due to insufficient source information; a large proportion of their associated genes are still unknown. One way to circumvent the difficulties in the lack of source information is to integrate available external information by using one of up-to-date integration or fusion methods. However, if one wants a disease network placing huge emphasis on the original source of data but still utilizing external sources only to complement it, integration may not be pertinent. Interpretation on the integrated network would be ambiguous: meanings conferred on edges would be vague due to fused information.

**Methods:**

In this study, we propose a network based algorithm that complements the original network by utilizing external information while preserving the network’s originality. The proposed algorithm links the disconnected node to the disease network by using complementary information from external data source through four steps: anchoring, connecting, scoring, and stopping.

**Results:**

When applied to the network of metabolic diseases that is sourced from protein-protein interaction data, the proposed algorithm recovered connections by 97%, and improved the AUC performance up to 0.71 (lifted from 0.55) by using the external information outsourced from text mining results on PubMed comorbidity literatures. Experimental results also show that the proposed algorithm is robust to noisy external information.

**Conclusion:**

This research has novelty in which the proposed algorithm preserves the network’s originality, but at the same time, complements it by utilizing external information. Furthermore it can be utilized for original association recovery and novel association discovery for disease network.

## Background

The amount of information on disease-disease association has been ever increasing over the last decade and the source of information also has been diversified from multi-levels of genomic data to clinical data, such as copy number alteration at the genomic level, miRNA expression or DNA methylation at the epigenomic level, protein-protein interaction at the proteomic level, disease comorbidity at the clinical level, and etc. [1–4].

One of the most effective ways to describe disease-disease association is by constructing a disease network, which consists of nodes and edges, representing diseases and disease-disease relations, respectively [5, 6]. In a disease network, the concept of disease-disease association (i.e., edges) varies depending on the source of information that the network utilizes. Many researches have been conducted using various sources of data. In Goh et al. [7], the authors created a disease network based on gene-disease associations by connecting diseases that are associated with the same genes. It had further developed in Zhou et al. [8] which constructed a diseases network by using disease-gene information and disease-symptom information. Lee et al. [9] constructed a network in which two diseases are linked if mutated enzymes associated with them catalyze adjacent metabolic reactions. While these researches are based on genomic data, there are also other researches that utilize clinical data for associated disease concerning patient records. In Hidalgo et al. [10], authors constructed a disease network, which reflects information of two co-occurred diseases, by utilizing clinical records of 13,039,018 patients. The authors utilized prevalence of two diseases co-occurring in a patient for edges. On the other hand, Žitnik et al. [11] is a research that uses both genomic and clinical data. In Žitnik et al. [11], the authors integrated data on disease-gene association, disease ontology, drugs and genes so that they could utilized such information to deduce disease-disease associations. So far, we see that most of these researches only utilize a single source of data to find disease-disease associations. On the other hand, if diverse and heterogeneous sources of data are available, there also have been network-wise approaches to integrate multiple disease networks for inferring associations between diseases [3, 12–15].

**Fig. 1:**
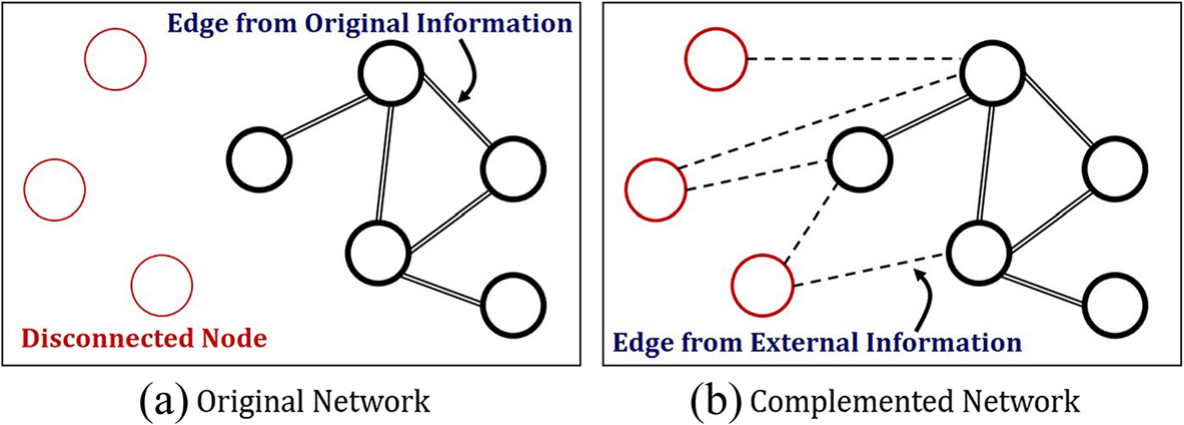
Proposed Method: **a** original network with disconnected nodes, and **b** complemented network that links the disconnected nodes to the connected network through newly found edges using external information

However, if one wants a disease network placing huge emphasis on a particular source of data but still utilizing other sources only to complement the original source, which researches above can be applied to it? For example, if we were to target drug discovery or to reposition by using disease network, the one constructed with protein information would be more preferred [16, 17]. On the other hand, if physicians were to treat a patient, they would prefer a disease network constructed with comorbidity information based on prevalence of diseases. If, however, there are losses or deficiencies of information in each original source, what would we do? In such a case, disease-disease associations cannot be defined, resulting in a disconnected network. See Fig. 1(a). If external source of data is usable, we could integrate the original network and the external network in a network-wise fashion by using one of up-to002Ddate integration methods [3, 12, 14, 18]. But interpretation on the results would be ambiguous: meanings conferred on edges would be vague in the resulting disease network.

This motivates the present research. In this paper, we propose an algorithm that preserves the network’s originality, but at the same time, complements it by utilizing external information. We denote the proposed algorithm as CLASH which abbreviates complementary linkage with anchoring and scoring for heterogeneous data. An original disease network is constructed from PPI information as in Goh et al. [7] and Zhou et al. [8]. And then, CLASH is applied to the network in order to link disconnected nodes to the network through newly found edges using external information. In the complementing process, clinical comorbidity information is used as external source of information. The resulting network is called as a complemented disease network. See Fig. 1(b).

The remainder of the paper is organized as follows. Section 2 introduces CLASH in length. Section 3 provides the experimental results on validity and utility of CLASH by applying it to metabolic disease group. Section 4 presents conclusion.

## Complementary linkage with anchoring and scoring for heterogeneous data

Disease network is a graph, *G* = (*V*, *W*), that describes connection between diseases with nodes and edges. In a disease network, a node denotes a disease and an edge denotes disease-disease association. Here, disease-disease association is a value obtained by calculating similarity between two diseases based on their shared genes (or proteins) and co-occurrence information through clinical trials. On the graph, similarity between two diseases are assigned with a weight value on the edge and higher of its value implies higher association between two diseases. In our study, the disease network is constructed using shared proteins: a disease vector has *n*-dimensional protein vector, and the similarity between two diseases are calculated with cosine similarity between disease vectors. If all disease gets connected to more than one edge, the disease network becomes a connected graph. On the other hand, if a disease is left to be disconnected from the network due to lack of disease-disease association with other diseases, it becomes impossible to deduce any inference about the disease from the network.

**Fig. 2:**
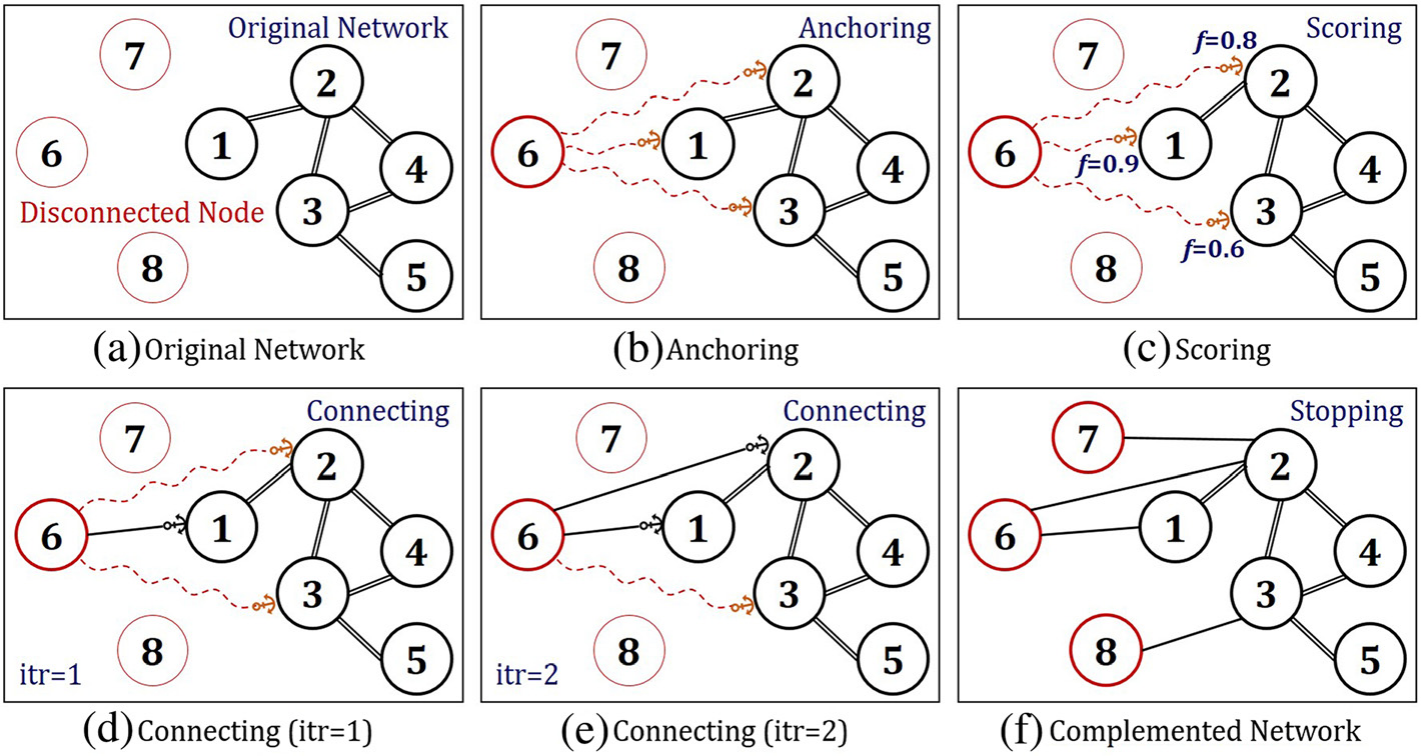
Schematic description of CLASH Algorithm

To circumvent the difficulty, we propose an algorithm for linking the disconnected node to the disease network by using complementary information from external data source, CLASH. The method is composed of four steps, anchoring, connecting, scoring and stopping. Figure 2 presents each step, beginning with a graph of eight nodes of which five are connected and three are disconnected.

### Anchoring

At the anchoring step, disconnected nodes are initially connected to the network (i.e., disconnected nodes drop their anchor to the connected graph). During the process, disconnected nodes must select a node to drop their anchor by utilizing possible external data source. Here, external data source is information unsuitable or less preferred, for purpose or usage of the proposed network. Thus, it is information that is not mainly used for constructing the network, but can be utilized to supplement the network. Figure 2(b) describes an anchoring step of a disconnected node, *v*_6_, to the connected graph of five nodes. Based on external data source, the fact that *v*_6_ is related to {*v*_1_, *v*_2_, *v*_3_} allows us to initially connect *v*_6_ to associated nodes. These associated nodes are defined as candidate nodes.

### Scoring

The scoring step allows disconnected nodes to select connectable nodes from anchored nodes through scoring. In this paper, we utilize the Semi-Supervised Learning (SSL) algorithm. The way it works is that given a connected graph, the SSL computes *f*-score for each labeled node. See Appendix. In the present study, the label of disconnected node is set to ‘1’, and ‘0’s for others. The *f*-score increases with stronger connectivity of associated edges and the number of edges [19–21]. In addition, the higher the *f*-score implies the higher similarity to the labeled node. Figure 2(c) shows the result of scoring step of a disconnected node, *v*_6_, on candidate nodes, *v*_1_, *v*_2_, *v*_3_. The *f*-scores for given nodes are {0.9, 0.8, 0.6}, respectively.

### Connecting

**Fig. 3:**
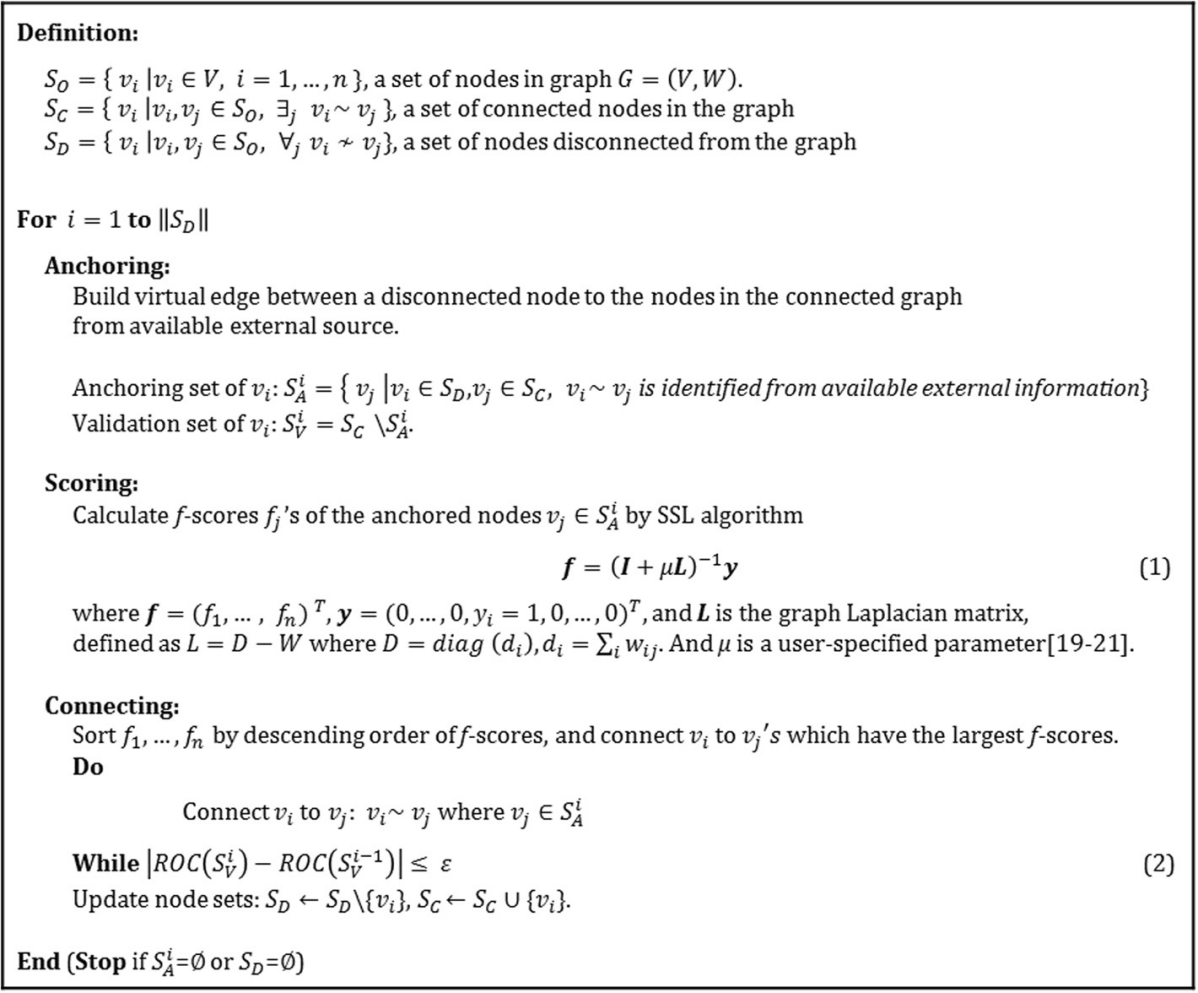
Pseudo Code of CLASH Algorithm

At the connecting step, disconnected nodes connect to the graph based on scoring results. The order of connection is determined based on the *f*-scores on candidate nodes. Higher the *f*-score means higher the priority in the order of connection. (If the *f*-scores of candidate nodes are the same, then they are connected to the graph with the same priority.) Newly formed edges through connection can cause disturbances (sometimes severe disturbances) on the network. Because severe disturbances could cause the original network to lose its property, there needs to be a standard that could determine the connection with certainty. In this research, we provide such standard due to its principle of preserving network properties and utilizing external data source. The preservation of network’s property can be measured through performance of the network whenever a new edge is formed between a disconnected node and a candidate node. Performance of network is measured on validation nodes, which excludes disconnected nodes and candidate nodes. Under the condition that the network’s performance stays within certain range (denoted by ϵ in (2) in Fig. 3), we then allow additional edges to be formed. If a change in network performance after connection is trivial, it implies that a newly connected node does not incur unexpected perturbation in the original network, thus preserving the original property of network. Figure 2(d) shows a candidate node, *v*_1_, connects to *v*_6_ due to its higher *f*-score compared to other candidate nodes {*v*_2_, *v*_3_}. At this point, the validation nodes are {*v*_4_, *v*_5_}. The connection is finalized since the change in additional/pre-post performance of the edge is within a certain range. After the first connection, we proceed to another candidate node, *v*_2_, which has the second largest *f*-score. Figure 2(e) shows the disconnected node, *v*_6_, making final connections to two of the candidate nodes, {*v*_1_, *v*_2_}, out of three candidate nodes that had been anchored.

### Stopping

The proposed algorithm stops when there are no more disconnected nodes, or external data or the performance of the network decreases. Figure 2(f) shows a network in which all the disconnected nodes, *v*_6_, *v*_7_, *v*_8_, are connected through previous steps.

The pseudo-code for the proposed algorithm is presented in Fig. 3.

## Experiments

### Data

The proposed algorithm was applied to the metabolic disease group. Demographically, metabolic diseases are widespread among people and show increasing rate in recent years. In up-to-date genome researches or molecular biology, however, it is difficult to trace *disease-protein* associations for metabolic diseases. This means that in researches that construct diseases network based on genome or protein information, it is also difficult to trace *disease-disease* associations for metabolic diseases. For example, in Goh et al. [7], it shows that there are almost no connections between metabolic disease nodes in human disease network, which is significantly different from nodes for cancer that have dense connections in the network. Thus, we chose metabolic diseases to construct a denser disease network by supplementing connections through CLASH. To construct a metabolic disease network, a list of diseases was obtained from Medical Subject Headings (MeSH) of the National Library of Medicine [22]. When considering up to the second level of the taxonomy, there are 302 descriptors for metabolic diseases out of 27,149 listed diseases. For the nodes, we acquired 53,430 data points on disease-protein associations. From the obtained set of data, we have selected and utilized 181 metabolic diseases and 15,281 proteins that were eligible to construct the disease network. The edge weights were calculated with cosine similarity between 15,281 dimensional disease vectors. We denote this network as the *original disease network*. For external data sources that could be used to complement the original disease network, we used comorbidity information reported on clinical literatures. Comorbidity addresses the concomitant occurrence of different medical conditions or diseases, usually complex and often chronic, in the same patient [23, 24]. In order to acquire external data source, text mining was conducted on 1,000,254 medical literatures from PubMed. From this point onward, we define *complemented disease network* as the resulting disease network complemented with comorbidity information through CLASH. Table 1 summarizes the source and type of data used in our experiment.

**Table 1:** Data sources for metabolic diseases, proteins, disease-protein associations, comorbidity

	Disease	Original source data		External source data
		Protein	Disease-Protein association	Comorbidity literature
Number of data	181 out of 302	15,281 out of 30,634	53,430 relations	6518 out of 1,001,254
Sources	Medical subject headings 2014	Comparative Toxicogenomics Database (CTD) Genetic Association Database (GAD) Online Mendelian Inheritance in Man (OMIM) The Pharmacogenomics Knowledge Base (PharmGKB) Therapeutic Target Database (TTD)		PubMed (05-01-31 ~ 15-03-31)

### Experimental settings

First, we have performed verification tests to see how the proposed algorithm, CLASH, complements the network. To carry out the tests, we gave artificial damages to the original network, allowing CLASH to recover the damaged network and to construct the complemented disease network. More specifically, we randomly chose and deleted 20, 40, 60 and 80 %, of the edges from the original diseases network and specified each resulting network as ‘*%-damaged original network’*. (For our convenience, ‘0 %-damaged original network’ is denoted as the *reference network*.) After constructing the complemented disease networks from each levels of damage, we compared them to each of the %-damaged original networks. Second, the overall performances would increase if we add further information from extra data. However, this would only happen if the extra source of data is useful to complement the original source of data. Therefore, to further clarify the validity of CLASH, we performed additional experiments comparing effects of noise data when employed to complement the %-damaged original network. They are denoted as noisy networks. To measure the network’s performances, we used SSL algorithm on prediction problems on possibly co-occurring diseases in the case when there is an outbreak of a certain disease [19]. *Leave-one-out* validation method is used [25]. The *f*-scores for all diseases are calculated by (1) except for one target disease. Then, the ROC was obtained by comparing *f*-scores and PubMed Literatures: presence (‘1’) or absence (‘0’) of PubMed literatures is used as a standard for disease association. For 181 diseases, the ROC was similarly calculated. The whole experiment was repeated 10 times.

## Results and Disscussion

### Results for validity of CLASH

**Fig. 4:**
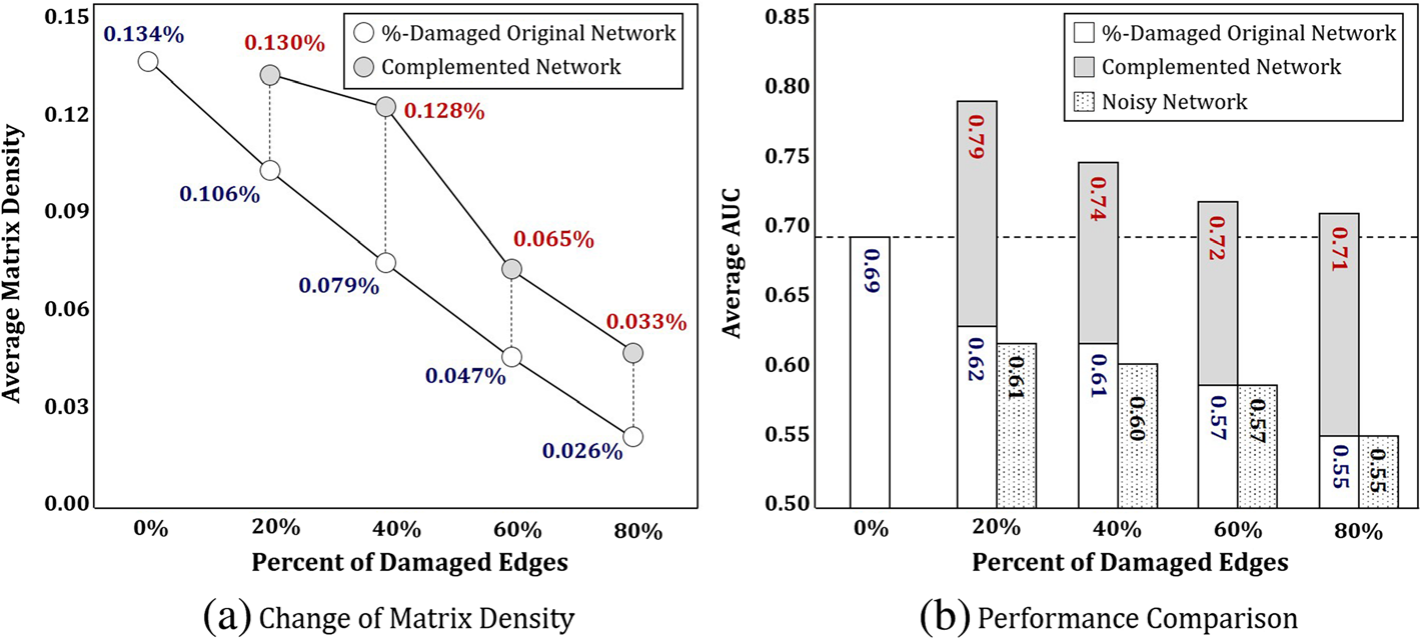
Results for Complementing Ability of CLASH: **a** shows that the proportion of edges have been recovered by 18 %, on average. **b** shows that CLASH improves AUC performance up to 0.79. The *p*-values for statistical tests for pairwise comparison between %-damaged original network and complemented network are 0.0002, 0.0001, 0.0002 and 0.000, respectively. On the other hand, CLASH is robust to noise: the noisy networks incurred insignificant degradation or no change in performance to %-damaged networks, preserving the original information

Figure 4(a) presents network density that depicts proportion of edges, which had been recovered through CLASH. It shows that, regardless of the degree of damages, by utilizing external data sources, the proportion of edges have been recovered by 18 %, on average. In case of 20 %-damaged network, 97 % of edges were recovered when comparing with those of reference network (97 % = (0.130/0.134) × 100 %.) Also, it is interesting to see that it is possible to recover severely damaged edges that had been deleted by 80 %. Fig. 4(b) shows comparisons of AUC performances of damaged network and complemented network. From the bar chart on 80 %-damaged network, we can see that CLASH improves the performance up to 0.71 (lifted from 0.55). Considering that the performance of reference network was 0.69, it can be inferred that CLASH has led to improvement in AUC even in the most severely damaged network. For other damaged networks, the comparisons can be similarly interpreted. On the other hand, the noisy networks incurred insignificant degradation or no change in performance to %-damaged networks. (The amount of noisy edges corresponds to those of complemented edges for %-damaged networks.) This shows that CLASH is robust to noisy external source data and preserves the original information.

### Result for utility of CLASH

**Fig. 5:**
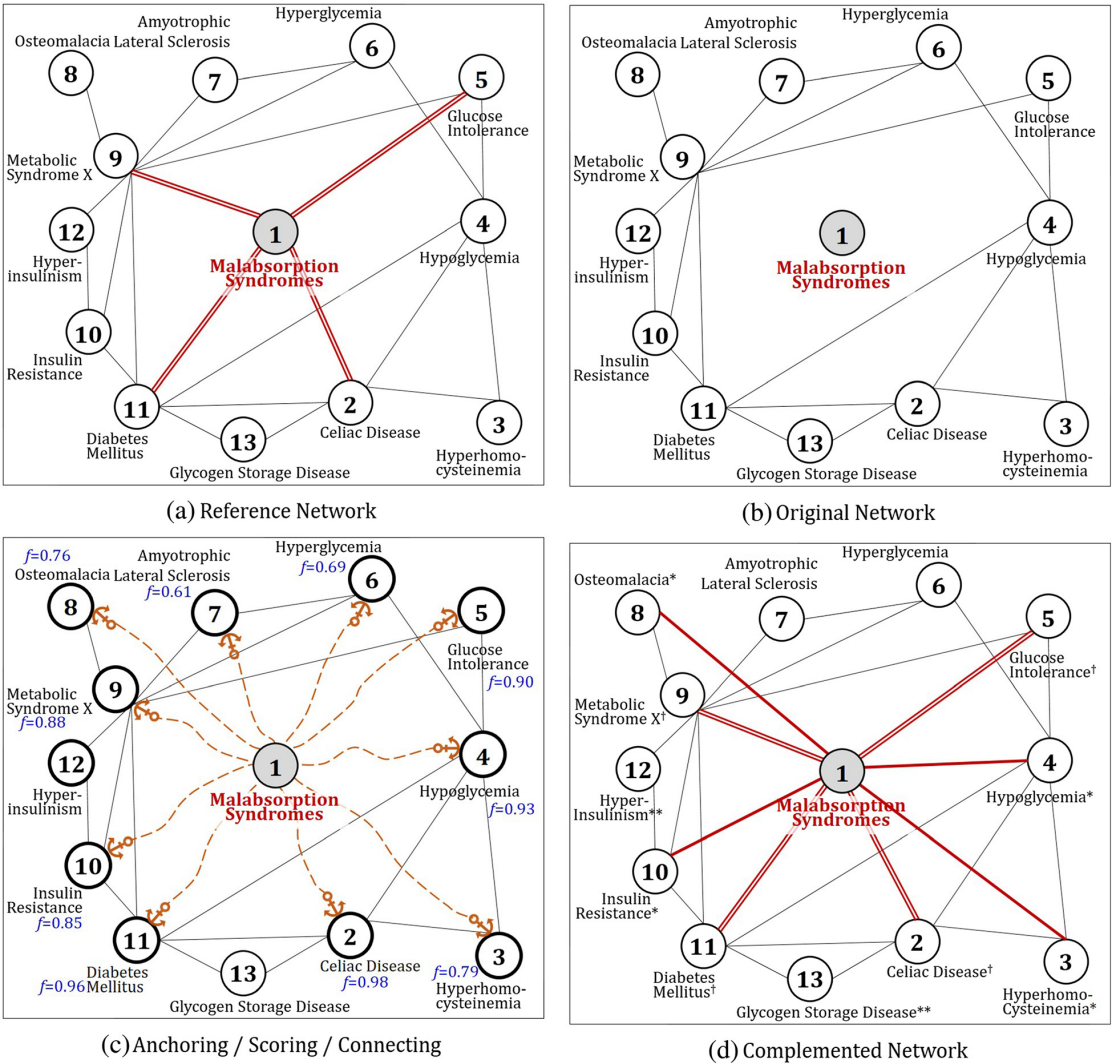
Utility of CLASH by demonstrating the process for the malabsorption syndrome: CLASH algorithm complements the network with four recovered edges and four newly found ones. Therefore, *malabsorption syndrome* extends its associations with more diseases, *hyperhomocysteinemia*, *hypoglycemia*, *osteomalacia* and *insulin resistance*, apart from the originally connected four diseases. Single solid lines refer to extended edges and double lines refer to original edges. Also notations ‘^†^’, ‘*’ and ‘**’ denotes associated diseases via PPI, PubMed and multiple paths involving more than one edge, respectively

In this section, we show utility of CLASH by demonstrating its process and typical results for a case disease. Malabsorption syndrome was selected as a target disease out of 181 metabolic diseases. Malabsorption syndrome refers to a wide variety of frequent and uncommon disorders of the process of alimentation in which the intestine’s ability to absorb certain nutrients, such as vitamin B12 and iron, into the bloodstream is negatively affected [26, 27]. Fig. 5 presents step-by-step process of CLASH for the target disease. Figure 5(a) shows a reference network of 13 disease nodes which simplifies the whole network of 181 diseases. In the figure, malabsorption syndrome (node 1) has four connections with *celiac disease*, *glucose intolerance*, *metabolic disease X* and *diabetes mellitus* (node 2, 5, 9, 11, in due order.) The four edges were purposely deleted to show if CLASH successfully recovers the original ones and further compliments the network with new edges from external knowledge found from PubMed comorbidity literatures. This is shown in Fig. 5(b), the original network. Figure 5(c) briefly describes anchoring, scoring and connecting: firstly, the node of malabsorption syndrome anchors at 10 nodes (See anchored diseases [28–37]) which includes the four nodes of the originally associated (node 2, 5, 9, 11) and six nodes of the newly found (node 3, 4, 6, 7, 8, 10). Among them, eight nodes are finally connected which have the highest values of *f-score* after dropping out two nodes with the lowest scores, node 6 and 7. Figure 5(d) presents the complemented network of four recovered edges and four newly found ones. Solid single line in the network refers to the former and double-line denotes the latter. Consequently, we see that malabsorption syndrome extends its associations with more diseases, *hyperhomocysteinemia*, *hypoglycemia*, *osteomalacia* and *insulin resistance* (node 3, 4, 8, 10), apart from the originally connected four diseases shown in Fig. 5(a).

**Fig. 6:**
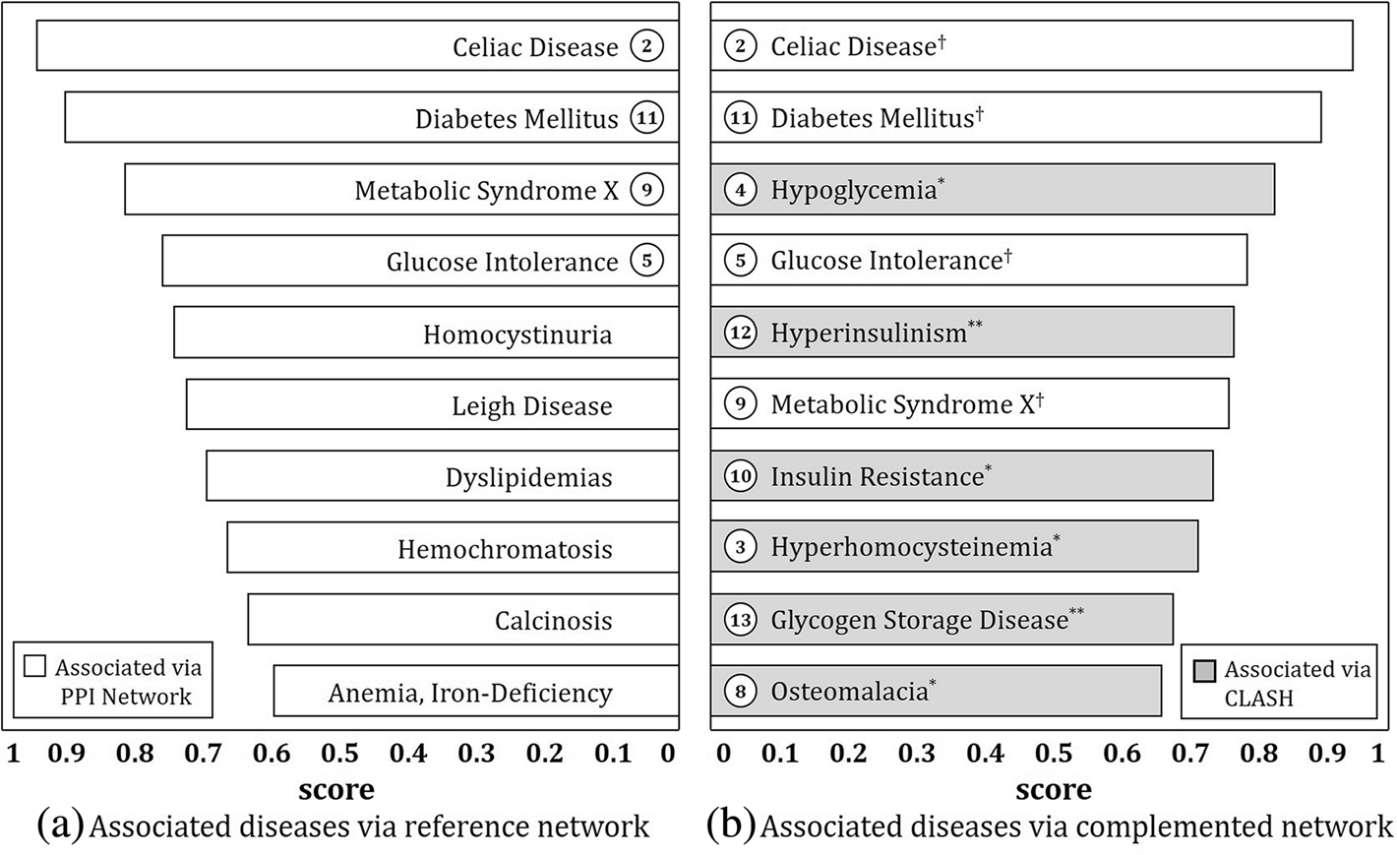
Top tier ranked up to 10th associated diseases with malabsorption syndrome: Notations ‘†’, ‘*’ and ‘**’ are identical to those in Fig. 5

To validate the utility of newly found edges, we performed disease scoring on reference network in Fig. 5(a) and complemented network in Fig. 5(d), and then compared the top tier ranked up to 10th associated diseases from each network. Figure 6 presents a comparison of disease list obtained from results of reference network and complemented network. Figure 6(b) shows that *celiac disease*, *glucose intolerance*, *metabolic disease X* and *diabetes mellitus* are highly ranked. If we compare these diseases with those connected to *malabsorption syndrome* in Fig. 5(d) (node 2, 5, 9, 11), we get an interesting result in which all these diseases are also included in the disease list. On the other hand, it is also notable that four diseases, *hyperhomocysteinemia*, *hypoglycemia*, *osteomalacia* and *insulin resistance*, that are associated with newly found edges in Fig. 5 (node 3, 4, 8, 10) are included in the list as well. From the results of Figs. 5 and 6, we see that CLASH is able to preserve the originality of the disease network built from PPI information, but at the same time, complements it by utilizing PubMed comorbidity literatures.

In a similar manner, an experiment has been carried out on 181 diseases (Supplemental materials http://www.alphaminers.net.). Table 2 illustrates results for 10 diseases. The first 5 diseases, similar to malabsorption syndrome, are artificially disconnected diseases from the original network of 181 diseases while the last 5 diseases are real disconnected diseases that does not contain any PPI information (not valid).

**Table 2:** Top tier ranked up to 10th associated diseases

Target Disease	Associated via Reference Network		Associated via Complemented Network	
Celiac Disease	Diabetes Mellitus Metabolic Syndrome X Glucose Intolerance Hyperinsulinism Hyperlipidemias	Calcinosis Dyslipidemias Hyperhomocysteinemia Anemia, Iron-Deficiency Homochromatosis	Diabetes Mellitus^†^ Metabolic Syndrome X^†^ Glucose Intolerance^†^ Hyperinsulinism^†^ Congentital Hyperinsulinism**	Malabsorption Syndromes* Hypoglycemia** Dyslipidemais^†^ Hyperglycemia** Hyperhomocysteinemia†
Lactose Intolerance	Diabetes Mellitus Metabolic Syndrome X Glucose Intolerance Celiac Disease Dyslipidemias	Hyperinsulinism Amyotrophic Lateral Sclerosis Insulin Resistance Hyperlipidemias Glucose Metabolism Disorders	Mucolipidoses* Celiac Disease* Glycogen Storage Disease* Metabolism, Inborn Errors* Malabsorption Syndromes*	Diabetes Mellitus^†^ Hypoglycemia** Hyperinsulinism** Glucose Intolerance** Hypokalemia*
Hypophosphatasia	Metabolic Syndrome X Diabetes Mellitus Glucose Intolerance Metabolic Diseases Dyslipidemias	Amyotrophic Lateral Sclerosis Diabetes, Gestational Hyperlipidemias Hyperinsulinism Calcinosis	Acidosis, Renal Tubular* Zellweger Syndrome* Celiac Disease* Peroxisomal Disorders** Refsum Disease*	Metabolism, Inborn Errors* Adrenoleukodystrophy* Homocystinuria** Diabetes Mellitus^†^ Phenylketonurias*
Refsum Disease	Zellweger Syndrome Peroxisomal Disorders Chondrodysplasia Punctata, Rhizomelic Adrenoleukodystrophy Homocystinuria	Porphyrias Protoporphyria, Erythropoietic Hyperhomocysteinemia Diabetic Ketoacidosis Lipid Metabolism, Inborn Errors	Neuronal Ceroid-Lipofuscinoses* Lipidoses* Peroxisomal Disorders^†^ Zellweger Syndrome^†^ Diabetes Mellitus*	Leigh Disease* Glycogen Storage Disease* Adrenoleukodystrophy^†^ Malabsorption Syndromes* Glucose Intolerance**
Fanconi Syndrome	Hypophosphatemia Glycogen Storage Disease Hypercalcemia Glucose Intolerance Metal Metabolism, Inborn Errors	Osteomalacia Xanthomatosis, Cerebrotendinous Diabetes Mellitus Calcinosis Metabolic Syndrome X	Celiac Disease* Glycogen Storage Disease^†^ Diabetes Mellitus^†^ Carbohydrate Metabolism, Inborn Errors* Leigh Disease*	Diabetes, Gestational* Glucose Intolerance^†^ Hyperinsulinism* Hypoglycemia* Congenital Hyperinsulinism**
Menkes Kinky Hair Syndrome	N/A		Congenital Disorders of Glycosylation* Hyperglycinemia, Nonketotic* Zellweger Syndrome* Peroxisomal Disorders** Hepatolenticular Degeneration*	Refsum Disease** Acidosis, Lactic* Albinism* Mitochondrial Myopathies* Adrenoleukodystrophy**
Pyruvate Carboxylase Deficiency Disease	N/A		Acidosis, Renal Tubular* Carbohydrate Metabolism, Inborn Errors* Hyperglycinemia, Nonketotic* Maple Syrup Urine Disease* Glycogen Storage Disease*	Amino Acid Metabolism, Inborn Errors* Renal Aminoacidurias** Metabolism, Inborn Errors* Acidosis, Lactic* Pyruvate Metabolism, Inborn Errors*
Rothmund-Thomson Syndrome	N/A		DNA Repair-Deficiency Disorders* Celiac Disease* Metal Metabolism, Inborn Errors** Acidosis* Xanthomatosis, Cerebrotendinous**	Hypercalcemia* Skin Diseases, Metabolic* Amyloidosis, Familial* Achlorhydria** Osteomalacia**
Sphingolipidoses	N/A		Neuronal Ceroid-Lipofuscinoses* Lipidoses* Carbohydrate Metabolism, Inborn Errors* Diabetes Mellitus* Peroxisomal Disorders*	Zellweger Syndrome** Glycogen Storage Disease* Adrenoleukodystrophy* Refsum Disease* Glucose Intolerance**
Alkaptonuria	N/A		Carbohydrate Metabolism, Inborn Errors* Glycogen Storage Disease* Zellweger Syndrome* Metabolism, Inborn Errors* Peroxisomal Disorders**	Amino Acid Metabolism, Inborn Errors* Refsum Disease* Adrenoleukodystrophy** Diabetes Mellitus* Lipid Metabolism, Inborn Errors*

Notations ‘^†^’, ‘*’ and ‘**’ are identical to those in Fig. 5

Through results from the experiment, we verified usefulness and effectiveness of CLASH, which uses both original and external data source to find diseases that could co-occur with target diseases.

## Conclusion

The research proposes an algorithm, also known as CLASH, which complements or strengthens connections between diseases in a disease network. The proposed algorithm is useful when the original disease network is incomplete and when supplementary information on disease association is available. The verification process for CLASH has been done by applying the algorithm on metabolic diseases. The original disease network was constructed based on PPI information. And through CLASH, disconnected edges were complemented or strengthened by supplemental information obtained from PubMed comorbidity literatures. In the experiment on validity, CLASH not only successfully recovered purposely deleted edges but also improved their performances: It showed full recovery of 20 % damaged edges and an increase of AUC performance from 0.69 to 0.79. In the experiment on utility, the research illustrates how to utilize CLASH through the toy example: In the case of malabsorption syndrome as the target disease, it delineates the process of finding a list of diseases that could co-occur with the target disease. Similar results are also shown with other metabolic diseases.

This research has novelty in following aspects. CLASH is a methodology that preserves the network’s originality, but at the same time, complements it by utilizing external information. CLASH has different utility than other methods that integrate multiple data sources in a network-wise fashion. It puts more emphasis on one data source than others: To complement disease-gene information (from biology) with comorbidity information (from medicines), or oppositely, to complement comorbidity information with disease-gene information. Examples of former usage can be found in drug discovery/repositioning in pharmacology while an example of latter usage is inferring disease co-occurrence when practicing. Moreover, these usages are topics for further researches.

## Appendix

Graph-based Semi-Supervised Learning

Disease network is a graph, *G* = (*V*, *W*), that describes connection between diseases with nodes and edges. In a disease network, a node denotes a disease and an edge denotes disease-disease association. Given a disease network, graph-based Semi-Supervised Learning (SSL) is employed to calculate the scores when a target diease is given. In the present study, the target disease is labeled as ‘1’, and other diseasese are labeled as ‘0’ (unlabeled). With this setting on a disease network, SSL provides the scores for diseases, in terms of *f*-score. The algorithm is summarized as follows, and more details can be found in [19–21].

In graph-based SSL, a connected graph *G* = (*V*, *W*) is constructed where the nodes *V* represent the labeled and unlabeled data points while the edges *W* reflect the similarity between data points. In binary classification problem, given *n*(=*n*_*l*_ + *n*_*u*_) data points from the sets of labeled ${S}_L=\left\{{\left({x}_i,{y}_i\right)}_{i=1}^{n_l}\right\}$ and unlabeled ${S}_U=\left\{{\left({x}_j\right)}_{j = {n}_l+1}^n\right\},$ the labeled nodes are set to *y*_*l*_ ∈ {−1, + 1}, while the unlabeled nodes are set to zero (*y*_*u*_ = 0). However, for scoring problem in the proposed algorithm, the *n*_*l*_ nodes are set to a unary label *y*_*l*_ ∈ {1} while the unlabeled *n*_*u*_ nodes are set to zero (*y*_*u*_ = 0). Resulting the learning process is to assign scores ***f***_***u***_^***T***^ = (*f*_*nl* + 1_, …, *f*_*n*_)^*T*^ on nodes *V*_*U*_. The edges between the two nodes *v*_*i*_ and *v*_*j*_. are measured by the Gaussian function

$$ {w}_{ij}=\left\{\begin{array}{cc}\hfill { \exp}^{- dist\ \left({v}_i,\ {v}_j\right)/{\sigma}^2}\ \hfill & \hfill if\ i\sim j\hfill \\ {}\hfill 0\hfill & \hfill otherwise\hfill \end{array}\right. $$

where *i* ~ *j* indicates that the two nodes are connected, and the value of the similarity is represented by a matrix *W* = {*w*_*ij*_}. Then the label information can propagate from labeled node *v*_*i*_ to unlabeled node *v*_*j*_ when the value of *w*_*ij*_ is large, their outputs are lely to be close. The algorithm will output an *n*-dimensional real-valued vector *f* = [*f*_*l*_^*T*^ *f*_*u*_^*T*^] = (*f*_1_, …, *f*_*l*_, *f*_*l* + 1_, …, *f*_*n* = *l* + *u*_)^*T*^. There are two assumptions: a *loss function* (*f*_*i*_ should be close to given label of *y*_*i*_ in labeled nodes) and *label smoothness* (overall, *f*_*i*_ should not be too different from *f*_*i*_ for the neighboringes.hese assumptions are reflected in the value of ***f*** by minimizing the following quadratic function:

$$ \underset{f}{ \min }\ {\left(f-y\right)}^T\left(f-y\right)+\mu {f}^TLf $$

where $\boldsymbol{y}={\left[{y}_1, \dots,\ {y}_{n_l},\ 0, \dots,\ 0\right]}^T$ and the matrix *L*, which is known as the graph Laplacian matrix, is defined as *L* = *D* − *W* where *D* = *diag*(*d*_*i*_), ${d}_i={\displaystyle \sum_i}{w}_{ij}$. The parameter *μ* trades off loss and smoothness. Thus, the solution of this problem becomes

$$ f={\left(I+\mu L\right)}^{-1}y $$
